# Enhancing Intradermal Delivery of Lidocaine by Dissolving Microneedles: Comparison between Hyaluronic Acid and Poly(Vinyl Pyrrolidone) Backbone Polymers

**DOI:** 10.3390/pharmaceutics15010289

**Published:** 2023-01-14

**Authors:** Delly Ramadon, Lissa Florencia Putri Sutrisna, Yahdiana Harahap, Kurnia Sari Setio Putri, Fathin Ulayya, Pietradewi Hartrianti, Qonita Kurnia Anjani, Ryan F. Donnelly

**Affiliations:** 1 Faculty of Pharmacy, Universitas Indonesia, Depok 16424, Indonesia; 2 School of Life Sciences, Indonesia International Institute for Life Sciences, Jakarta 13210, Indonesia; 3 Medical Biology Centre, School of Pharmacy, Queen’s University Belfast, 97 Lisburn Road, Belfast BT9 7BL, UK

**Keywords:** dissolving microneedles, hydrosoluble, intradermal drug delivery, lidocaine, local anesthesia, PVP, hyaluronic acid

## Abstract

Lidocaine hydrochloride (LiH), an amide-type local anesthetic agent, is commonly used in dermatological procedures. LiH is categorized as a BCS (biopharmaceutics classification system) class III group, which has high solubility and poor permeability. It should be noted that, in this context, LiH is intended as a local anesthetic, so the level of LiH in systemic circulation should be minimized to avoid toxicity and unwanted side effects such as hypotension and bradycardia. This study aimed to formulate and evaluate LiH-loaded dissolving microneedles (DMNs) with different polymer bases. Moreover, an in vitro permeation study using Franz diffusion cells and in vivo study were also performed. LiH-loaded DMNs were prepared using polymer groups of poly(vinyl pyrrolidone) (PVP-K30) and hyaluronic acid (HA). DMNs were created using the micro-molding method with centrifugation. The formulations selected based on the evaluation were F3 (HA 10%) and F5 (PVP-K30 25%). Based on the in vitro permeation study, the amount of drug permeated and deposited in the skin at F3 (HA 10%) was 247.1 ± 41.85 and 98.35 ± 12.86 μg, respectively. On the other hand, the amount of drug permeated and deposited in the skin at F5 (PVP-K30 25%) was 277.7 ± 55.88 and 59.46 ± 9.25 μg, respectively. Our in vivo drug-permeation study showed that only one rat from the PVP-K30 polymer group—with a concentration of 150.32 ng/mL—was detected on rat plasma. Therefore, LiH can be formulated into a DMN and can be deposited in the skin with a safe concentration of the drug permeating into systemic circulation.

## 1. Introduction

Dermatological procedures are associated with pain and discomfort in patients. Patient complaints, such as procedural pain, stress, and fear of needles, become clinical considerations and concerns when performing dermatological procedures [1]. Pain management is crucial to improve recovery and minimize healthcare costs. Treatment and management of pain are important and expensive issues; the high cost of pain has been extensively reported as a significant contributor to healthcare costs [2]. A more efficient pain management strategy is needed without compromising patient quality [3].

One strategy that can be employed to minimize pain is the use of local anesthetic agents. Local anesthetics are safer when compared to other anesthetic methods because of their minimal drug dose and fast recovery [4]. Lidocaine hydrochloride (LiH) is one of the most widely used local anesthetic therapeutic agents [5,6]. LiH is an active substance that is categorized as a BCS (biopharmaceutics classification system) class III drug, which has high solubility (0.054 g/mL) with poor permeability (LogP = 2.3) [7]. Generally, this drug is administered in the form of an injection via the intramuscular, intravenous, and subcutaneous routes, which may provide higher bioavailability [8]. However, the injection route has some disadvantages, such as pain when administered, risk of infection, local irritation, trypanophobia (excessive fear of needles), the need for medical personnel who are experienced in drug administration, problems related to medical waste, and high storage costs [4,9,10]. Furthermore, one of the most crucial drawbacks of these parenteral routes is the high exposure to systemic circulation, which increases the potential for systemic side effects. This is not negligible in the case of LiH. Since LiH is intended for local anesthetic effects, the systemic exposure of lidocaine should be minimized to avoid toxicity and unwanted side effects, such as hypotension and bradycardia [11,12]. Toxic effects may occur if the plasma concentration of LiH is more than 6 mg/L [11]. These factors could provide impetus to develop and formulate different polymer bases to create more promising formulations and minimize drug permeations into circulation in order to prevent the toxicity.

An alternative strategy, performed to overcome the problems mentioned above, particularly in dermatological procedures, is to formulate LiH in other dosage forms, such as creams, gels, and sprays [13]. Delivering LiH via skin local delivery may avoid its systemic toxicity, which common after parenteral administration. However, the drug must be able to cross the stratum corneum, the main barrier to the skin, to have a local anesthetic effect [14,15]. LiH has been marketed in the form of an anesthetic cream, such as 5% EMLA™ [4,16]. Nevertheless, LiH should be combined with prilocaine to improve its penetration [17]. LiH has also been formulated into different types of conventional topical dosage forms, namely gel and spray. However, these approaches still produce very limited amounts of drug that penetrate into the skin due to the low permeability of LiH [18]. LiH itself has low skin permeability, and conventional products of this local anesthetic agent are not effective in penetrating intact skin [19]. Therefore, a different approach is required to improve LiH skin penetration and minimize its side effects.

Microneedles (MNs) are a dosage form with a transdermal and intradermal delivery system in the form of non-invasive technology that can bypass the stratum corneum layer to deliver drugs into deeper skin layers. MNs are applied to the skin surface, creating microscopic channels through which the drug diffuses into dermal microcirculation [20,21]. Currently, MNs for anesthetic therapy have been developed by various researchers as they show promising benefits for this purpose. Due to the superiority of MNs that can pass through the stratum corneum, they may allow for an accelerated onset of therapy [4,16,22]. This is evidenced by several studies that continue to be developed. From a geometric point of view, the shape of the needle in the previous study was conical with a total of 60 needles [4]. The first study succeeded in formulating and conducting an in vivo permeation test for MNs coated with LiH. The coated MNs were square pyramids composed of approximately 316 microneedles (height of 500 μm and a tip-to-tip needle spacing ~550 μm) [16]. Although the formulation was successful in delivering the drug in less than 5 min (tissue concentrations of lidocaine and prilocaine were approximately 50 ng/mg each), the amount of drug delivered was insufficient to exert an anesthetic effect. This is due to the small MNs-coated surface. Moreover, coated MNs have problems related to low biocompatibility and hazardous waste [4].

In the current investigation, LiH was included into DMN (LiH-DMN) to develop a promising formulation able to minimize permeation into the systemic circulation, thus minimizing systemic toxicity. As the backbone polymer of LiH-DMN, two polymers were tested separately: PVP-K30 and hyaluronic acid. PVP is an inert, non-toxic, thermostable, pH stable, biocompatible, and biodegradable polymer, and it can assist in the encapsulation of hydrophilic and lipophilic drugs [23]. Hyaluronic acid is an unbranched linear polysaccharide with repeating disaccharide units containing D-glucuronic acid and N-acetyl-D-glucosamine linked by beta-1,4-glycosidic bonds [24]. These two polymers are commonly used in the medical and pharmaceutical fields due to their good physical, chemical, and biocompatibility properties. In this study, the two polymers were compared to see which was preferable for use as the backbone polymer of DMN. In terms of manufacturing techniques, the micro-molding technique was used in this study because it is suitable for mass production at a relatively low cost [25]. This research used pyramidal-shaped needles, with total of 100 needles, which had the potential to provide better penetration and drug release. The formulation of DMNs was evaluated and characterized in terms of their physical and chemical properties. In addition, the permeation of LiH in DMNs was also tested in an in vitro test using a Franz diffusion cell and an in vivo study using an animal model of the Sprague Dawley strain rat. The samples of in vitro and in vivo studies were then analyzed using high-performance liquid chromatography (HPLC).

## 2. Materials and Methods

### 2.1. Materials

Lidocaine hydrochloride (LiH) was given by PT Phapros, Indonesia. Hyaluronic acid (HA) (Mw ~60 kDa) was purchased from Shandong Topscience Biotech Co., Ltd., Rizhao, China. Polyvinyl pyrrolidone K30 (PVP-K30) (30–40 kDa) was purchased from BASF, Hong Kong. All chemical and reagents used in this study were analytical grade. All solvents used for analytical study were HPLC-grade.

### 2.2. Methods

#### 2.2.1. Fabrication and Physical Evaluation of Lidocaine Hydrochloride-Loaded Dissolving Microneedles (LiH-DMNs)

LiH with a concentration of 5% (*w*/*w*) was dissolved in distilled water. The hyaluronic acid (HA) or polyvinyl pyrrolidone K30 (PVP-K30) at different concentrations (Table 1) was added to the drug solution to form a polymer–drug mixture and then poured into a silicone micro-mold (10 × 10 pyramidal needles with 600 μm needle height, 200 μm needle base, and 500 μm needle pitch). The polymer–drug mixture (both in the needles section and baseplate) was centrifuged at a rotation speed of 3500 rpm for 30 min [26]. Then, the mixture was dried for 24 h at room temperature (28 ± 2 °C). After drying, the DMN was then carefully removed from the mold and stored in a desiccator under vacuum for further use. Each DMN was evaluated for its physical appearance and dimensions using a light microscope. Dimensional measurements observed include needle height, base width, and distance between needles [9].

#### 2.2.2. Mechanical Strength of LiH-Loaded DMNs

The mechanical strength of DMNs was evaluated during a compression test using a TA.TX2 texture analyzer (Stable Microsystems, Haslemere, UK) with compression mode [27]. The DMNs were attached to the cuboidal probe on the texture analyzer and the probe was programmed to move vertically downwards at a speed of 0.5 mm/s with a force of 32 N onto 8 layers of Parafilm M^®^. Then, the MN was held for 30 s. The needle height was then observed and compared before and after compression using a digital light microscope. MN height reduction percentage was calculated by Equation (1), where Hb and Ha are the height of the needles before and after compression, respectively [9].

(1) $$Height\;Reduction\;\left(\%\right)=\frac{Hb-Ha}{Hb}\times 100\%$$

#### 2.2.3. Loss of Mass

The mass loss of water during the drying process was calculated based on the mass comparison before (*m_b_*) and after drying (*m_a_*). The percentage of total water lost after drying was calculated using Equation (2) [26].

(2) $$\%Loss\;on\;Drying=\frac{m_{b}-m_{a}}{m_{b}}$$

#### 2.2.4. Insertion Study

Eight layers of Parafilm M^®^ were used for the insertion study, which simulated an artificial skin model [28]. DMNs were then inserted into 8 layers of Parafilm M^®^ using a texture analyzer with a compression mode of 32 N. After compression, each layer of Parafilm M^®^ was observed under a light microscope, and the number of holes formed was recorded. The penetration percentage of the layer can be calculated by Equation (3) [26,28]:

(3) $$\%Holes\;Created\;on\;Each\;Layer=\frac{Holes_{n}}{Holes_{total}}\times 100\%$$

where *Holes_n_* and *Holes_total_* are the number of holes created in each layer of Parafilm M^®^ and number of needles in each array, respectively.

#### 2.2.5. Ex Vivo Skin-Dissolution Study

An ex vivo skin-dissolution study was carried out using rat skin. First, the rats were euthanized by injecting an excessive dose (120 mg/kg) of anesthetic agent ketamine intraperitoneally [29]. The skin was carefully shaved using a hair clipper. In order to remove the remaining hair, hair removal cream was used by applying it to the skin, and we waited for 5 min [9]. Then, the skin was wiped with a paper towel until there was no remaining cream on the skin. The skin was rinsed with phosphate buffer solution (PBS) pH 7.4 and blotted by a paper towel to ensure that it was dry. The rat skin on the abdomen was excised and the subcutaneous fat was carefully removed. The skin was then equilibrated in PBS (pH 7.4) and stored at −20 °C before use. Prior to the study, the skin was equilibrated in a PBS (pH 7.4) and carefully shaved. Each DMN was observed under a light microscope to record its initial height and capture images. Then, each DMN was applied to the prepared skin pieces. DMNs applied to the skin were then removed from the skin at 0, 2.5, 5, 10 min, and until the needles were completely dissolved [9].

#### 2.2.6. Determination of Drug Content in the Needles

The assay was carried out by calculating the LiH content in the whole MN, the baseplate, and the needle section. Each part of the needles and baseplate was separated using a scalpel [9]. Prior to analysis, it was necessary to carry out dilutions to ensure that the analyte concentration examined was within the range of the calibration curve. Furthermore, 200 µL of each diluted solution was then analyzed using high-performance liquid chromatography (HPLC) with a method that has been validated based on the standards set by the European Medicines Agency (EMA) and Food and Drug Administration (FDA) [30,31], which is explained in Section 2.2.10.

#### 2.2.7. Stability Study of LiH in DMN

LiH-loaded DMNs were stored in a tightly closed plastic container at room temperature 28 ± 2 °C for 35 days, and changes in drug levels were observed on day 1 and day 35 using the assay method described in Section 2.2.10. The LiH content in the DMN was analyzed to determine chemical stability during storage [4].

#### 2.2.8. In Vitro Permeation Study

The two most promising formulations with different polymer were tested for drug permeation in an in vitro study. The in vitro permeation study was carried out using Franz diffusion cells with Sprague Dawley rat skin as the membrane and PBS (pH 7.4) as the receiving fluid in the receptor compartment medium. The skin was attached to the donor compartment using cyanoacrylate glue with the stratum corneum layer facing up in the donor compartment. The DMNs were placed in the center of the skin using an applicator and pressed for 30 s by pushing the flat end of the syringe plunger against the surface of the DMN baseplate. Then, a metal weight (5.0 g) was placed on top of the DMNs to hold them during the study. A Parafilm M^®^ layer was then placed over the donor chamber and on the receptor arm to prevent loss of the medium. The temperature was maintained at 37 ± 1 °C and the receptor compartment (15 mL) was stirred at 600 rpm using a magnetic stirrer (10 mm × 4 mm). Samples (200 µL) were taken through the sampling port at 15, 30, 45, 60 min, and then continued at 2, 4, 6, 8, 10, 12, and 24 h. Phosphate buffer saline (PBS pH 7.4) was immediately replaced into the receptor compartment at each sampling point [9,32]. After 24 h, the skin was removed from the compartment of the Franz diffusion cell. Using a scalpel, extraction of the drug remaining in the skin was undertaken by removing the skin sample from the donor compartment and cutting it into small pieces with an area of 1 cm^2^. Then, the scalpel was also rinsed with PBS (pH 7.4) and deposited into an Eppendorf^®^ tube, which was then sonicated (bath sonicator) in 5 mL of PBS (pH 7.4) at 37 °C for 24 h using an ultrasonic bath (Branson 3200, City of Alameda, CA, USA). Samples were filtered using a 0.45 µm syringe filter before being analyzed [33]. All samples were diluted appropriately and analyzed using the HPLC method, as described in Section 2.2.10.

#### 2.2.9. In Vivo Permeation Study

The animal models used in this study were male Sprague Dawley rats aged 8–10 weeks which had been acclimatized for 7 days before being tested. Acclimatization was carried out by separating rats into small cages (4 rats per cage) divided into 2 groups (n = 4 per group), each of which was a group with hyaluronic acid and PVP-K30 polymer-based DMN. The animal study protocol was approved by the Health Research Ethics Commission, Faculty of Medicine, University of Indonesia Cipto Mangunkusumo Hospital (RSCM) with the ethical approval number: No.KET-385/UN2.F1/ETIK/PPM.00.02/2022 on 18 April 2022.

Before the treatment, the rats were anesthetized intraperitoneally using the anesthetic agents with a dosage of 80 mg/kg ketamine and 16 mg/kg xylazine [30]. A blank patch made of wrapping a square-shaped Micropore^®^ tape was made utilized to hold the DMNs. Then, two DMNs (LiH 2.10 mg/DMN) were applied to the dorsal skin using thumb pressure. Each blank patch was placed on the surface of the DMN base, followed by the last layer using kinesiology tape on the back of the rat to avoid the movement of the DMNs that had been applied to the rat. Blood samples were obtained by taking the tail vein with a maximum volume of 200 µL at 15, 30, 60, 90, 180, 360, and 720 min. Blood was taken through the tail vein of rats into a vacutainer with a volume of 0.5 mL containing the anti-coagulant heparin [4,9].

#### 2.2.10. Analytical Method of LiH and Chromatographic Condition

LiH analysis was carried out using an HPLC system (Shimadzu, Japan) with a UV-Vis detector (UV- Vis SPD-20A) and autosampler. The samples were separated using a Xbridge C18 column (250 × 4.6 mm i.d. with 5 µm particle size) with a UV detector set at 230 nm, and the mobile phase used was phosphate-buffer-acetonitrile (74:26) pH 4.5, flow rate was 0.8 mL/min, and column temperature of 25 °C. This chromatographic condition was referring to a previous study that has been optimized and validated as per the Food and Drug Administration (FDA) and European Medicine Agency (EMEA) bioanalytical method validation guidelines [30,31,34].

#### 2.2.11. Statistical Analysis

All statistical analyses in this study were performed using GraphPad Prism version 8.3. The data obtained from the study are shown as average ± SD processed with Microsoft Excel Office 365 and GraphPad Prism programs. One-way ANOVA and *t*-test were used to examine significant differences between formulations. A *p* value < 0.05 indicates a significant difference.

## 3. Results and Discussion

### 3.1. Fabrication and Physical Evaluation of Lidocaine Hydrochloride-Loaded Dissolving Microneedles (LiH-DMNs)

The concentration LiH was chosen in accordance with the concentrations available on the market, which was in the range of 0.5–5% *w*/*w*. DMNs were formulated using several excipients with different concentrations, as shown in Table 1.

**Figure 1:**
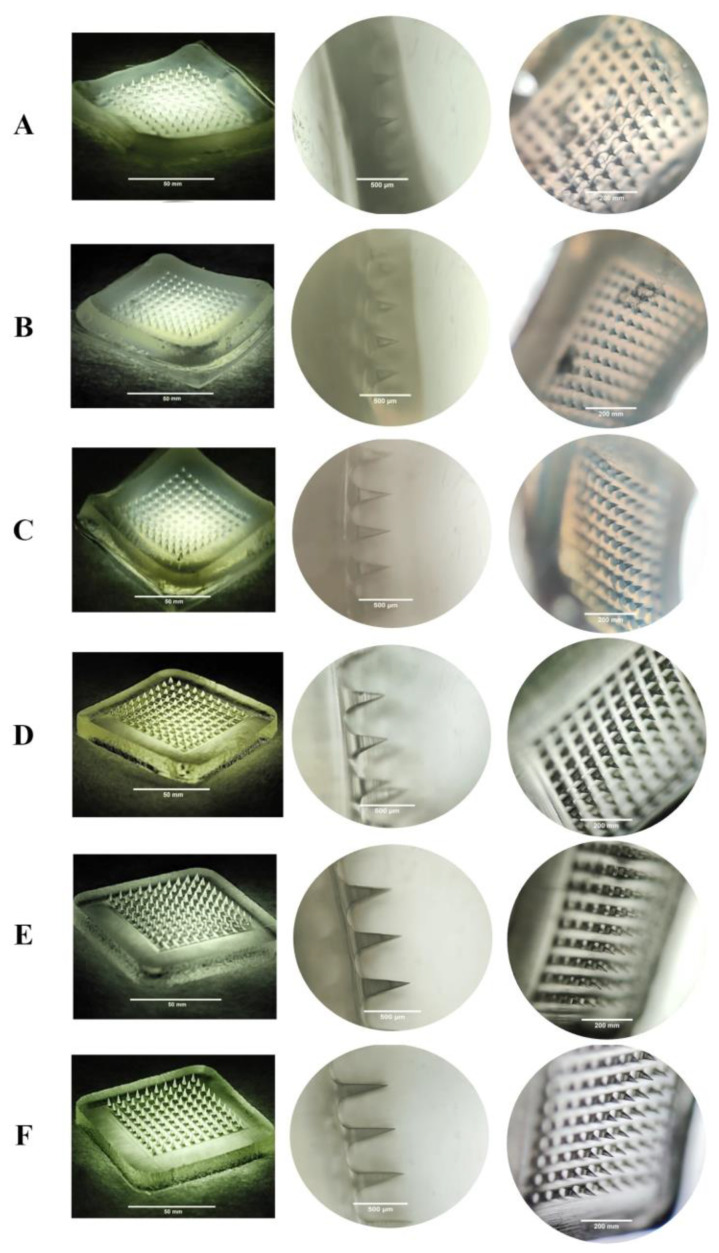
Physical evaluations using light electron microscope (10×, 40×, and 100×) of (**A**) F1, (**B**) F2, (**C**) F3, (**D**) F4, (**E**) F5, and (**F**) F6.

The dimensions of the DMNs (needles height, width, and distance between needles) were measured using a light microscope (Leica EZ4W, Germany) with a magnification of 40× and 100×, which are listed in Figure 1. Based on the physical evaluation summary listed in Table 2, F1 showed air bubbles, the surface of the needles was jagged, and the needles’ height was 303.35 ± 0.63 µm. In terms of F2, there were damaged needle parts, and the needles’ height was 354.004 ± 1.05 µm. The needles’ surface of F3 was slightly wavy, had no air bubbles, and the needles’ height was 410.282 ± 0.95 µm. Therefore, F1 and F2 were not used in the subsequent evaluation because they did not meet some physical parameters. These unfulfilled physical parameters are related to the mechanical ability of the DMNs. The ability of DMNs to be inserted into the skin is a very important parameter because they must be able to penetrate the stratum corneum to deliver drugs [28]. Poor physical evaluation results, such as jagged and damaged needles, represent poor mechanical properties of the DMNs. F3 was used in the next evaluation because this formulation almost fulfilled all physical parameters. In the PVP-K30 polymer-based formulation—namely F4, F5, and F6—all three showed the required physical appearances, such as flat surface of baseplate and no bubbles [9]. The needle heights of F4, F5 and F6 were 524.343 ± 3.93 µm, 550.120 ± 0.31 µm, and 580.173 ± 0.69 µm, respectively. Therefore, all PVP-K30 polymer-based formulas were further assessed in subsequent evaluations.

The typical thickness of the stratum corneum is about 10 µm [21,35,36]. However, there are many factors that may influence the thickness of the stratum corneum, such as the location of the skin and skin hydration [36,37]. To illustrate this variability, it is important to note that the thickness of the stratum corneum on the palms of the hands can reach 400–600 µm, and that hydration can lead to a fourfold increase in thickness [20]. Based on this variability, the optimal needle height of DMNs is supposed to be more than 400 µm. In the HA-based polymer group, only F3 passed the criteria for physical evaluation. However, all formulations in the PVP group passed physical evaluation. Additionally, DMNs should not only be evaluated by their appearance, but they should also be strong enough to breach the skin, especially the stratum corneum layer [9].

### 3.2. Mechanical Strength of LiH-Loaded DMNs

**Figure 2:**
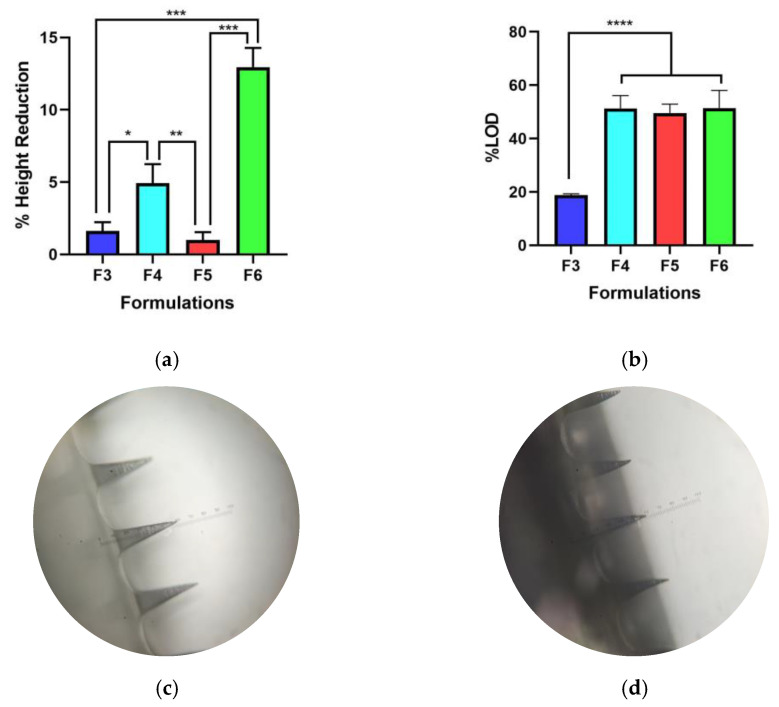
DMN evaluations of (**a**) mechanical strength and (**b**) loss on drying. Images of the F5 (**c**) before and (**d**) after the compression. The data presented are shown as mean + SD (n = 3; *: *p* < 0.05; **: *p* < 0.01; ***: *p* < 0.0005; ****: *p* < 0.0001).

The percentages of needle height reduction were determined using a texture analyzer with compression mode. Based on Figure 2a, all formulations exhibited strong mechanical characteristics and did not break when given a force of 32 N. Among the formulations tested, F5 showed the lowest mechanical properties. This result revealed that the optimum concentration of PVP to be used for LiH was up to 25% *w*/*w*. Increasing PVP concentration resulted in weaker mechanical properties of the prepared DMNs. Thus, F6 showed the highest percentage of height reduction at 12.96 ± 1.32% when compared to F3 (1.63 ± 0.60%), F4 (4.93 ± 1.32), and F5 (0.99 ± 0.55%) (*p* < 0.0005). The percentage of height reduction should not be more than 10% [9]. The results indicate that the percentage of PVP-K30 polymer can affect the strength of the needle. This is in accordance with the study conducted by Shim et al., who reported that excessive concentrations of PVP can affect needle strength, making it weaker in relation to skin penetration, the effectiveness of which is lowered due to increased hygroscopic properties and moisture binding due to the addition of PVP [38].

### 3.3. Loss of Mass

DMNs were weighed before and after drying in order to obtain the percentage loss on drying (LOD), as shown in Figure 2b. Based on the one-way ANOVA test of the PVP-K30 polymer group (F4 = 51.32 ± 479%; F5 = 49.56 ± 3.37%; F6 = 51.40 ± 6.60%), there was no significant difference in LOD percentage (*p* > 0.05). However, there was a significant difference when compared to the HA polymer group (*p* < 0.0001). This may be due to the presence of a carboxylate group in the HA structure that attracts water molecules through the formation of hydrogen bonds. Water forms hydrogen bonds between the carboxylic group of glucuronic acid and the acetamido group of N-acetyl-D-glucosamine. Positive dipoles in water are attracted to negatively charged carboxylic groups, whereas negatively charged oxygen in water is attracted to nitrogen groups in acetamide functional groups. Water can form bonds between each HA subunit; thus, HA is able to retain water molecules [39]. The percentage of mass reduction in the HA polymer group was not more significant than that in the PVP group. The percentage value of mass loss after drying needs to be considered, as the high-water content in the formula can affect mechanical strength, which in turn affects the ability of the needle to penetrate skin and the effectiveness of drug delivery [40,41].

### 3.4. Skin Simulation Insertion Study

**Figure 3:**
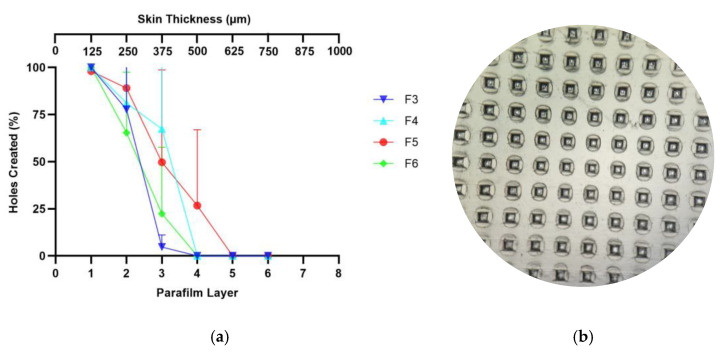
(**a**) Graph of the percentage of holes formed by each layer of parafilm. Data are represented as means + SD (n = 3). (**b**) The microscopic image of holes created on parafilm layer.

The ability of MNs to penetrate the skin is one of the important factors before they dissolve and release drugs [9]. Therefore, it was imperative to examine the penetrative ability of DMNs in the skin. In this study, an artificial skin model using eight layers of Parafilm M^®^ was used. Based on studies related to artificial skin models using Parafilm M^®^ conducted by Larrañeta et al. (2014), one layer of Parafilm M^®^ had a thickness of 126 ± 7 µm (28). To penetrate the stratum corneum layer, which is about 400–600 µm, the DMNs should be able to penetrate the fourth parafilm layer, which is equivalent to a thickness of 500 µm [9,20,21]. The data in Figure 3a,b show the holes created in each layer of Parafilm M^®^ when DMN was applied by a texture analyzer. The data revealed that all formulations tested could penetrate at least into the third layer of Parafilm M^®^. HA-based DMNs showed very poor penetration into the skin compared to other formulations. Although it has strong mechanical strength, the base of F3, which is quite wavy, allows it to be a weakness in the efficiency of needle penetration into the skin, which can only penetrate up to the third layer. On the other hand, the PVP-K30 group was able to penetrate up to the fourth layer of Parafilm M^®^. This is supported by a study conducted by Shim et al., which revealed that the formulation with the addition of PVP can increase the efficiency of needle penetration into the skin when compared to the formula without PVP [38].

### 3.5. Ex Vivo Skin-Dissolution Study

**Figure 4:**
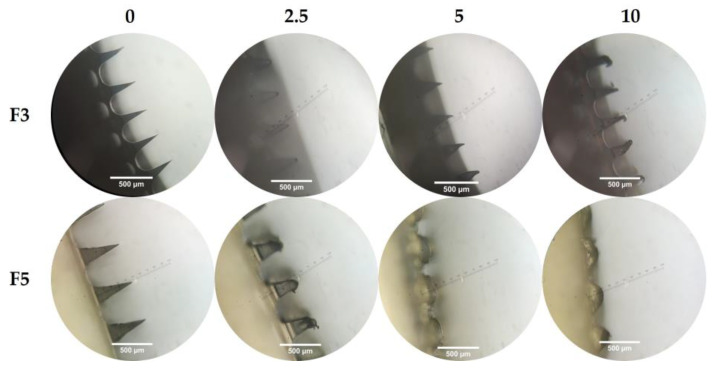
Dissolution of the needle of F3 and F5 in the skin with an interval of 0; 2.5; 5; 10 min (n = 3).

After establishing the mechanical strength and ability of the needle to penetrate the skin, the DMs were then tested regarding their dissolution in the skin. In this study, we found that the needles of F3 completely dissolved in 2 h and 10 min (Figure 4). Nevertheless, F5 showed a shorter duration, which was 20 min. This is in accordance with the study conducted by Shim et al., which showed that the higher the concentration of PVP in DMNs, the faster the dissolution rate [38,42]. On the other hand, high concentrations of HA polymer may lead to stiffer DMNs, which can also be used for sustained release delivery [9,43,44].

### 3.6. Determination of Drug Content in the Needles

Based on the determination of LiH content on day 0 (without storage), the percentage of LiH in F3 was in the range of 96.57–106.11%, whereas F5 was in the range of 100.12–103.81%. The LiH amount was also calculated on the needle section and the baseplate. In F3, the LiH amount on the needles’ section and the baseplate was 0.08 ± 0.15 mg and 2.16 ± 0.16 mg, respectively, whereas in F5, the LiH on the needle and base was 0.08 ± 0.09 mg and 2.08 ± 0.12 mg, respectively. Both F3 and F5 showed that the level of LiH in the DMN met the accuracy range for pharmaceutical dosage form, which is 90–110% [45].

### 3.7. Stability Study of Lidocaine

The chemical stability test aimed to determine the stability of LiH in the DMNs during storage by measuring the LiH content in the DMNs using HPLC. In this study, the stability test was carried out for 35 days. The LiH recovery in F3 on the 1st and 35th days was 100.22 ± 5.15% and 99.05 ± 4.96%, respectively. However, the LiH levels in F5 on the 1st and 35th days were 101.55 ± 1.97% and 100.29 ± 2.39, respectively. These data indicated that the levels of LiH in F3 and F5 on the 1st and 35th days showed no significant difference (*p* > 0.05). This result shows that LiH, which was formulated into a DMN dosage form, was stable in storage until at least 35 days. This is also in accordance with previous studies, where LiH made into DMN was stable in storage. Despite being stored under extreme temperature conditions for 6 months, the LiH content in the DMN remained at more than 90% [4].

### 3.8. In Vitro Permeation Study

**Figure 5:**
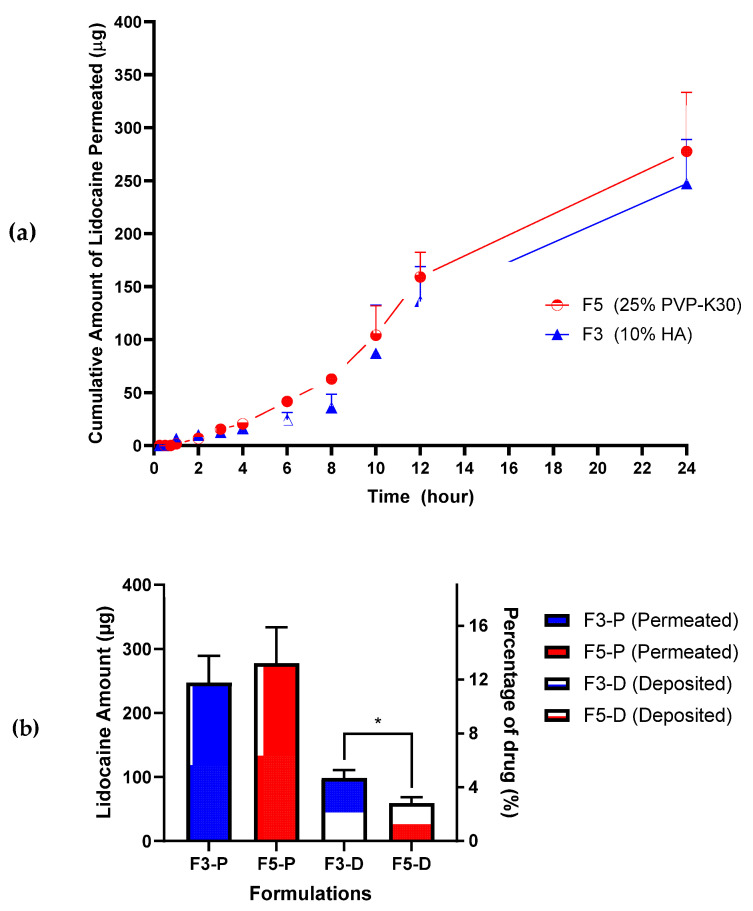
The LiH amount of (**a**) cumulative permeation in 24 h; (**b**) the comparison of the deposited and permeated based on in vitro study. Data are presented as means + SD (n = 3; *: *p* < 0.05).

Based on Figure 5a, the cumulative amount of LiH permeated in F3 and F5 was 247.1 ± 41.85 µg and 277.7 ± 55.88 µg, respectively. All LiH contained in the needles section from F3 and F5 was fully permeated (100%) in 24 h. The cumulative amount of LiH permeated in 24 h in F5 was slightly higher than F3. However, there is no significant difference in the data (*p* > 0.05). Based on Figure 5b, the amount of LiH deposited on the rat’s skin applied to DMN F3 and F5 was 98.35 ± 12.86 µg and 59.46 ± 9.25 µg, respectively.

It is worth noting that the mechanism of action of LiH as a local anesthetic is by blocking the conduction of sensory nerves to noxious stimuli—so that they do not reach the central nervous system—by binding to voltage-gated sodium channels on the excited membrane [46,47]. Therefore, LiH should be deposited in the dermis of the skin where nerve endings are located [48]. In addition, the cumulative amount of LiH permeated into the receptor compartment was quite high due to the more porous structure of the rat skin [21].

When comparing the amounts of LiH permeated from F3 (247.1 ± 41.85 μg) and F5 (277.7 ± 55.88 μg), we observed no significant difference (*p* > 0.05). However, there was a significant difference in the drug deposited in the skin after our in vitro release study (*p* < 0.05), where F3 (98.35 ± 12.86 μg) showed a larger amount of LiH deposited than F5 (59.46 ± 9.25 μg). F3 with an HA polymer base has the advantage of increasing skin hydration because of its humectant properties, which draw water from the atmosphere [49]. Skin hydration can lead to a fourfold increase in stratum corneum thickness [20]. Therefore, it will increase drug retention into the dermis layer and reduce drug permeation that enters systemic circulation [50]. Based on the data obtained, some LiHs on the baseplate can also be released into the skin. Therefore, the selection of DMNs with both the baseplate and the needles containing the drug (single layer) is a promising strategy, as it can provide a more effective anesthetic effect with a larger amount of drug loaded into DMNs.

### 3.9. In Vivo Permation Study

**Figure 6:**
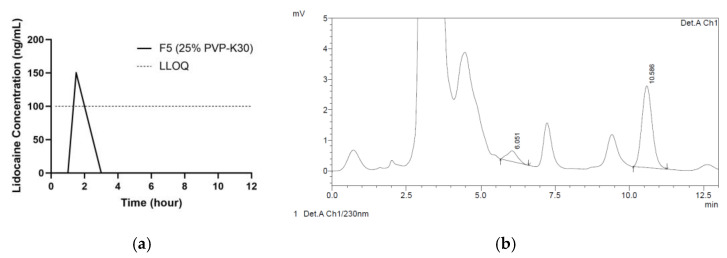
(**a**) The amount of LiH detected in one rat plasma at time point of 1.5 h from PVP group based on in vivo study. (**b**) Chromatogram of the plasma analysis following the application of F5 (LiH = 6.051 min).

In all rats allocated to F3 or F5, there was a small concentration of drug permeated into systemic circulation at 1.5 h, as depicted in Figure 6a. However, those concentrations were below the lower limit of quantification (LLOQ). Figure 6b shows the chromatogram of the plasma analysis following the application of F5 (LiH = 6.051 min). Therefore, only concentrations greater than LLOQ, which was 100 ng/mL, can be declared valid. In the DMN group that applied DMNs with F3, no lidocaine was detected that entered systemic circulation. The concentration of LiH permeated into systemic circulation, which was higher than the LLOQ (100 ng/mL), was only found in one among eight rats tested from the F5 group, with a concentration of 150.32 ng/mL. This concentration was detected in one rat from the DMN group that was applied with F5 (PVP-K30 based) at 1.5 h.

The sampling time was adjusted to the plasma half-life of LiH, which is 1–2 h, and refers to previous in vivo studies [4,11]. The results from our in vivo approach were in line with previous in vitro drug release studies. As LiH is intended for local anesthetic effects, its systemic circulating level should be minimized to avoid toxicity and unwanted side effects such as hypotension and bradycardia [11,12]. However, in formulation F5, the concentration of LiH in plasma detected (150.32 ng/mL) was still relatively safe, as toxic effects can occur if the concentration of LiH in plasma is more than 6000 ng/mL [11]. This systemic exposure of LiH is important to note in order to avoid any unwanted side effects. A previous study comparing the release of LiH in DMN dosage forms and anesthetic creams such as 5% EMLA™ cream (containing LiH and prilocaine) showed that the permeation into systemic circulation of 5% EMLA™ cream was significantly higher compared to the DMNs [4]. Therefore, the drug delivery of LiH in the form of DMNs can be a more effective strategy, as it is relatively simple yet painless to administer and avoids or minimizes unwanted side effects.

## 4. Conclusions

LiH was formulated into DMNs with different types of polymers. The PVP-based DMNs provide a relatively faster release compared to HA-based DMNs, which have a slower release but are able to deposit more LiH in the skin. DMNs can be a strategy to deliver drugs more effectively, safely, and without any addition of permeation enhancers that may lead to the high systemic exposure of LiH. Although PVP-based DMNs in this study showed a small amount of LiH permeated into the bloodstream, the amount detected was very small and still relatively safe. In terms of the future perspective, it is essential to perform an animal study to investigate the pharmacodynamics of manufactured LiH-loaded DMNs.

## Figures and Tables

**Table 1:** The composition of LiH-Loaded DMNs.

Formulations	Composition (% *w*/*w*)			
	LiH	HA	PVP-K30	Water
F1	5	2.5	-	Ad 100
F2	5	5	-	Ad 100
F3	5	10	-	Ad 100
F4	5	-	20	Ad 100
F5	5	-	25	Ad 100
F6	5	-	30	Ad 100

**Table 2:** Summary of Physical Evaluations.

Formulations	Physical Appearance Parameter						Decision
	Air Bubbles	Drug Precipitation	Breaking	Flat Baseplate	Optimal Needles Filling	Optimal Needle Heights	
F1	☓	✓	✓	☓	✓	☓	Discarded
F2	✓	☓	✓	☓	✓	☓	Discarded
F3	☓	☓	☓	☓	✓	✓	Selected
F4	☓	☓	☓	✓	✓	✓	Selected
F5	☓	☓	☓	✓	✓	✓	Selected
F6	☓	☓	☓	✓	✓	✓	Selected

## Data Availability

Not applicable.
